# Biofilms can act as plasmid reserves in the absence of plasmid specific selection

**DOI:** 10.1038/s41522-021-00249-w

**Published:** 2021-10-07

**Authors:** Henriette Lyng Røder, Urvish Trivedi, Jakob Russel, Kasper Nørskov Kragh, Jakob Herschend, Ida Thalsø-Madsen, Tim Tolker-Nielsen, Thomas Bjarnsholt, Mette Burmølle, Jonas Stenløkke Madsen

**Affiliations:** 1 Section of Microbiology, Department of Biology, University of Copenhagen, Copenhagen, Denmark; 2 Costerton Biofilm Center, Department of Immunology and Microbiology, University of Copenhagen, Copenhagen, Denmark; 3 Department of Clinical Microbiology, University Hospital of Copenhagen, Rigshospitalet, Copenhagen, Denmark; 4 Department of Veterinary and Animal Sciences, University of Copenhagen, Copenhagen, Denmark

**Keywords:** Biofilms, Microbial genetics, Molecular evolution

## Abstract

Plasmids facilitate rapid bacterial adaptation by shuttling a wide variety of beneficial traits across microbial communities. However, under non-selective conditions, maintaining a plasmid can be costly to the host cell. Nonetheless, plasmids are ubiquitous in nature where bacteria adopt their dominant mode of life - biofilms. Here, we demonstrate that biofilms can act as spatiotemporal reserves for plasmids, allowing them to persist even under non-selective conditions. However, under these conditions, spatial stratification of plasmid-carrying cells may promote the dispersal of cells without plasmids, and biofilms may thus act as plasmid sinks.

## Introduction

In bacteria, low diversity from clonal reproduction is overcome by their ability to exchange genetic information via horizontal gene transfer. Here, plasmids play a major role in microbial ecology and evolution by shuttling new and accessory gene functions both within and across microbial species. They allow bacteria to rapidly adapt to environmental stressors (e.g., antimicrobials^1,2^) and perform functional leaps to access new niches (e.g., pathogenicity^3^ or anoxygenic photosynthesis^4^). However, despite the benefits they confer, plasmid replication and gene expression utilizing host machinery can also be a metabolic burden in the absence of selective pressure for plasmid-encoded traits^5^. This may lead to plasmid extinction, as plasmid-free cells will eventually outcompete plasmid-carrying cells. However, this assumption does not hold true for environmental bacteria, where antibiotic resistance plasmids are found even when there are essentially no antibiotics present^6^. This could suggest that plasmids spread fast enough horizontally to establish themselves even without being selected for^7^. This is, however, still being debated^8^ as the conjugal transfer is relatively infrequent even under optimal laboratory conditions^9,10^. Therefore, understanding the factors that facilitate plasmid stability and maintenance in populations is of major interest. Molecular mechanisms that maintain plasmids within cells are known, such as toxin–antitoxin and partitioning systems, while co-evolution can reduce the burden the plasmid imposes on the host^5,10^; however, less is known about which environmental conditions are conducive for maintaining plasmids.

**Fig. 1:**
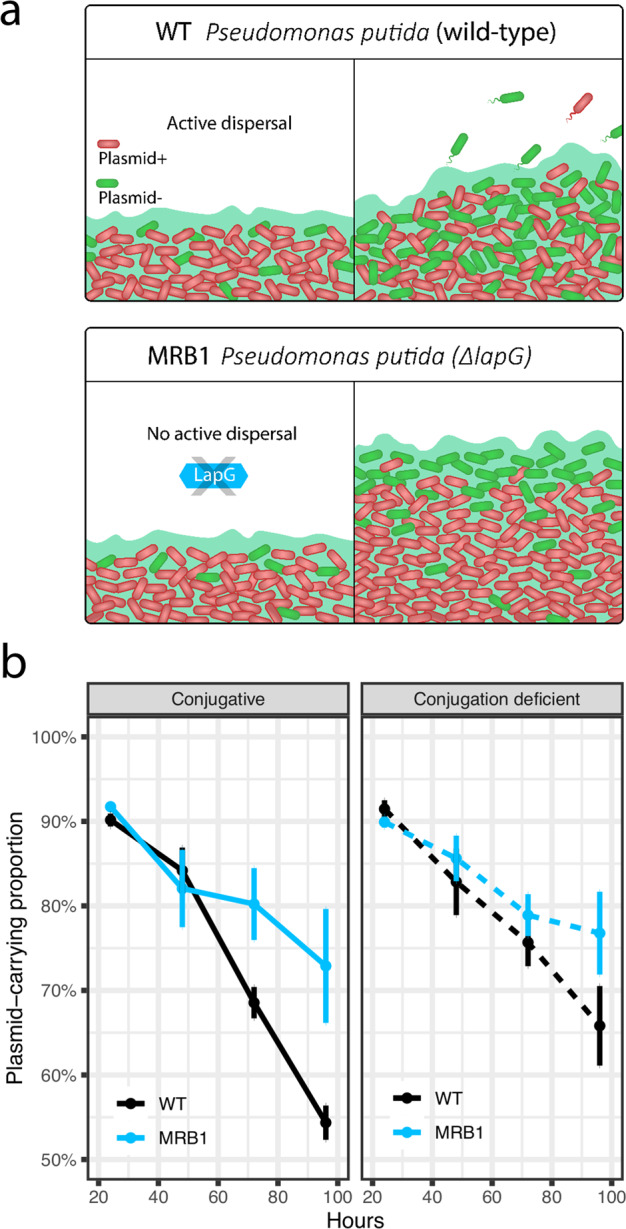
Plasmid maintenance in biofilms over time. **a** Schematic prediction of plasmid loss in biofilm. Dividing cells can lead to segregational plasmid loss-making biofilms act as a reserve for plasmids because of the presence of metabolically inactive cells. However, dispersal from the biofilm (WT) will influence the stability of the plasmid in the population over time. Here, we used an isogenic mutant MRB1, incapable of biofilm dispersal, to examine the influence of biofilm dynamics on plasmid maintenance. **b** Plasmid loss over time in biofilms. Plasmid loss in biofilm cultures of a dispersing (WT) and non-dispersing (MRB1) variant of *P. putida* monitored for 4 days. Error bars are standard error of the mean.

Here, we test if biofilms act as reserves for plasmids, ensuring their maintenance without plasmid-specific selection in line with a previous postulate^11^. This is a key question as biofilms, dense bacterial communities embedded in an extracellular polymeric matrix, are widely regarded as the dominant lifestyle of bacteria^12^. As a biofilm matures, physicochemical gradients are formed due to the spatial structure of these communities. For example, in surface-attached biofilms, the top layers nearest to substrate contain actively replicating cells, whereas the lower layers of a biofilm tend to consist of less active or dormant cells when the surface itself does not serve as a source of the substrate. Segregational plasmid loss occurs when bacterial cells divide and a copy of the plasmid is not passed to a daughter cell. Such plasmid loss is more likely to happen when cells divide rapidly, which bacteria do in the top layers of biofilms. Conversely, the less active or dormant bacteria in the biofilm have been speculated to act as reserves of plasmids. However, biofilms are dynamic communities where cells may disperse, actively or by sloughing^13^, which can influence their spatiality and thus maintain plasmids over time (Fig. 1). Here, we test the maintenance of plasmids in biofilms and planktonic environments and evaluate how spatiotemporal dynamics influence plasmid loss.

## Main text

Initially, we constructed a dual-labeled plasmid loss reporter system based on the conjugative plasmid pKJK5. A P_*A1O4O3*_*-gfp* fusion was inserted on the chromosome of the plasmid hosts, whereas P_*lpp*_-*mCherry* and *lacI*^*q*^, which represses GFP expression, were inserted on the plasmid. Therefore, mCherry is expressed only when the plasmid is present, whereas GFP is expressed only when the plasmid is lost. It has previously been shown that the pKJK5 plasmid conjugates in both planktonic and biofilm cultures^14,15^. This reporter system enabled spatial community analysis and made it possible to determine the rate of plasmid loss on a cellular level even if occurring at low frequency.

Here, *Pseudomonas putida* KT2442 and an isogenic mutant, MRB1, that forms biofilm but cannot actively disperse^16^, were used as our model to study the influence of biofilm formation and dispersal on plasmid stability. We expected that when the bacteria were unable to disperse from the biofilm, it would result in a higher retention rate of the plasmid (Fig. 1). Furthermore, two versions of the plasmid were used to evaluate the effect of conjugation on maintaining the plasmid; one that conjugates and another that has a highly reduced rate of conjugation^17^. To test if there was an effect of strain or plasmid variant, all pairs of associated plasmid-free and -carrying strains were grown in co-cultures (Supplementary Fig. 1a, b). Plasmid-free strains outcompeted plasmid-carrying strains (two-tailed Welch’s *t* test, *P* = 9.85 × 10^−16^) showing a cost to plasmid carriage. No effect of neither strain nor plasmid variant was found (two-way ANOVA and post hoc Tukey HSD test, *P* > 0.99).

Initially, plasmid loss was tested in biofilms grown in silicone tubes over 4 days. Planktonic cultures that were sub-cultured daily for 4 days were cultivated in parallel as controls. The spatially structured MRB1 biofilms, in which cells were incapable of active dispersal, had a higher retention rate of the plasmids compared to the wild-type (WT) (linear model and the emtrends function, *P* = 0.0239 for conjugative and *P* = 0.0055 for conjugation-deficient plasmid) (Fig. 1b). There was no apparent effect of conjugation in maintaining the plasmids which is noteworthy as biofilms have previously been shown to facilitate conjugation^18^. This underlines the need for a better understanding of how conjugation is affected by the spatiotemporal dynamics that occur during biofilm development. In planktonic cultures (Supplementary Fig. 1c), both versions of *P. putida* lost the plasmids to the same extent while being serial transferred for 4 days (linear model and the emtrends function, *P* = 0.37 for conjugative and *P* = 0.21 for conjugation-deficient plasmid) and the conjugation efficiency of the plasmids had no observable effect on their stability.

**Fig. 2:**
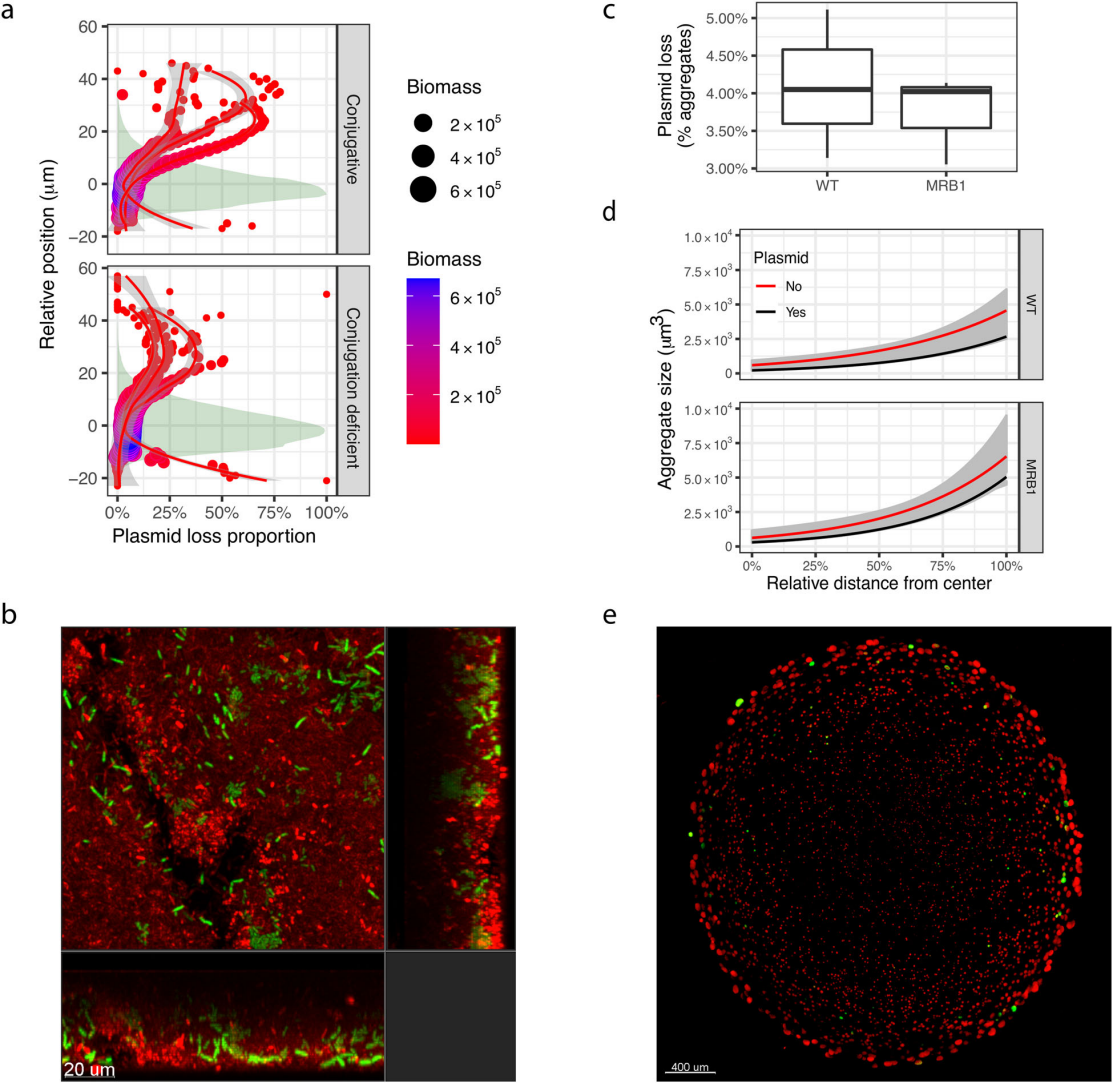
Spatial dynamics of plasmid maintenance in biofilms. **a** Plasmid loss in a flow cell. The proportion of cells without plasmids at different positions throughout the vertical axis of the biofilm of MRB1. The red lines are LOESS regressions of the plasmid loss weighted by the biomass, with a line for each replica. The shaded regions denote the total biomass distribution scaled such that max is 100%. Areas of points are proportional to the biomass. **b** Representative confocal image of flow-cell biofilm. Red cells carry plasmids. Green cells have lost the plasmid. The scale bar corresponds to 20 µm. **c** Plasmid loss in alginate beads. Ratio of cell aggregates without and with plasmids reflecting the rate of plasmid loss. Boxplot elements are: center line-median; box limits-upper and lower quartiles; whiskers-1.5 × interquartile range; points-outliers. **d** Aggregate size is shown as a function of the distance from the center of the alginate bead. Distances are normalized to the maximum distance for each bead. The line is a linear regression on log_10_ transformed aggregate sizes, shading is 95% confidence limits. **e** Representative confocal image of an alginate bead. Red cells carry plasmids. Green cells have lost the plasmid. The scale bar corresponds to 400 µm.

We hypothesized that there would be higher segregational plasmid loss in the top of the surface-attached biofilms than the lower layer because substrate gradients impact growth rates in these structured communities. This was examined by laser scanning microscopy (CLSM) on biofilms of MRB1 grown in flow cells. Here, higher plasmid loss was found in the top layers of the biofilms for the conjugative and conjugation-deficient plasmid (two-way ANOVA, difference between top and bottom layers *P* = 0.014, difference between plasmids *P* = 0.19), showing that there was a higher number of plasmid-free cells among these actively replicating cells (Fig. 2a, b).

To test if the observed difference in plasmid loss in WT and MRB1 biofilms was linked to dynamic remodeling of the biofilm structure caused by active dispersal of the WT, both *P. putida* WT and MRB1 were grown in alginate beads, where every single cell forms a defined microcolony. Alginate beads diminish the difference in biofilm formation between the two strains because dispersal is restricted, enabling directly comparable quantification of plasmid loss for both. As shown in Fig. 2c–e, similar levels of plasmid loss were observed for both strains (two-tailed Welch’s *t* test, *P* = 0.62), and the aggregates formed by plasmid-free cells were larger than those of plasmid containing cells (linear mixed-effect model on log-transformed aggregate sizes, *P* < 0.001). This confirmed that increased levels of plasmid loss were linked to the dynamic development of the biofilm structure. We found that plasmid-free cells grew faster than those with plasmids, implying that losing the plasmid provides a growth advantage in spatially structured communities when plasmid-encoded traits are not selected for.

Here, the occurrence of plasmid-free cells over time was comparable between planktonic (Supplementary Fig. 1c) and biofilm cultures (Fig. 1b). It has previously been argued that plasmid loss is more frequent in biofilms compared to planktonic cultures^18,19^. This might indeed be true in the regions of the biofilm where cells are actively dividing, and competition is fierce due to steep substrate gradients. Yet, when biofilms mature and some cells become inactive, they act as plasmid reserves because biofilms generally provide a protective environment for bacteria against threads such as e.g., grazing protozoa, phages, and antimicrobials^13^.

We find that biofilm dispersal by the WT strain led to increased plasmid loss. This is in agreement with plasmid retention being associated with bacteria with low metabolic activity. When cells disperse and become planktonic the substrate gradient is disrupted, leading to an increase of cellular activity in the newly exposed regions of the biofilm. Due to continuous dispersal in the wild-type biofilm, the fraction of actively growing bacteria (in the population consisting of biofilm and dispersed bacteria) is higher than the fraction of actively growing bacteria in the dispersal-deficient biofilm.

Interestingly, our findings, therefore, suggest that biofilms may act as plasmid sinks: The proportion of plasmid-free cells that are most likely to disperse from the top layers of the biofilms was higher than those of cells reserved in the deeper layers of the biofilms (Fig. 2a). This implies that the initial proportion of plasmid-carrying cells was higher when the biofilm was established but lower among the cells that disperse from the biofilm. So, whereas plasmid transfer appears to be facilitated during early biofilm establishment, and plasmids are maintained in maturing biofilms due to spatial reverberations, dispersal may represent a bottleneck for plasmids in the absence of plasmid-specific selection.

Our findings show that biofilms can act as reserves of plasmids even under non-selective conditions. In agreement, Bakkeren et al.^20^ recently showed that persister cells of *Salmonella enterica* serovar Typhimurium can act as long-lived reserves of resistance plasmids during infection.

Considering the ubiquity of biofilms and their protective effects on bacteria both in the environment and during infections^10^, it is very likely that biofilms are important reserves of plasmids under non-selective conditions.

## Methods

### Strains, plasmids, and growth conditions

The bacterial strains, plasmids, and their relevant characteristics are listed in Supplementary Table 1. The strains were grown in lysogeny broth (LB) (10 g L^−1^ tryptone, 5 g L^−1^ yeast extract, and 4 g L^−1^ NaCl) when needed, 1.5 g L^−1^ agar was added. Final concentrations of antibiotics used when required: 25 µg mL^−1^ kanamycin (Km), 60 µg mL^−1^ tetracycline (Tet), 20 µg mL^−1^ gentamicin (Gm). Cultures with plasmids used for starting experiments were always grown with appropriate antibiotics to prevent plasmid loss.

### Construction of strains and plasmids for detection of plasmid loss

*P. putida* KT2442 and *P. putida* MRB1 were both tagged with P_A10403_*gfp*mut3-*Gen*^*R*^ using the Tn7 system^21–23^. pMiB4 and pMiB8 are derivatives of the conjugative IncP-1 plasmid pKJK5 that was previously complemented with kanamycin resistance and constitutively expressed *lacI*^q^. Here, we inserted *mCherry* and *Gen*^*R*^ into *drfA1* of pMiB4 and pMiB8 using the λ Red recombination system^24^. Combining these plasmids with P_A10403_*gfp*mut3-tagged *P. putida* KT2442 and *P. putida* MRB1, enabled the detection of plasmid loss: cells with plasmids express plasmid-encoded mCherry, while chromosomal GFPmut3 is repressed by plasmid-encoded LacI^q^. Cells that become plasmid-free will start expressing GFP only, as plasmid-encoded mCherry and the LacI^q^ repressor are lost.

### Planktonic and biofilm plasmid loss detected with flow cytometry

The stability of both the conjugative and the conjugation-deficient plasmid was studied in planktonic and biofilm cultures to evaluate the effect of horizontal transfer on plasmid loss in the absence of plasmid-specific selection. The planktonic cultures were grown in tubes containing 5 mL of LB. From these cultures, 5 µL was transferred to new 5-mL LB tubes daily. The tubes were incubated at 24 °C and 250 rpm. To obtain the ratio of plasmid-free cells compared to plasmid-harboring cells, the samples were prepared by washing the cells in 0.9% saline solution (NaCl: 9 g L^−1^) three times before analysis on a FACSAria IIIu flow cytometer (Becton Dickinson Biosciences, San Jose, CA, USA).

The biofilm experiments were performed in silicone tubes with LB medium diluted ten times. Silicone tubes measuring 3 × 1 × 5 mm (Mikrolab Aarhus A/S) were used for biofilm growth in combination with an assortment of appropriate connectors and a 205 S/CA peristaltic pump (Watson Marlow). Overnight cultures were adjusted to OD_600_ of 0.01 prior to inoculation. Hereafter, the bacteria were allowed to attach to the flow cell for 2 h. After this, the flow was started at 2.5 mL h^−1^ so that non-attached cells were washed out of the system. Biofilms were grown at room temperature.

The biofilms were collected by harvesting three samples from one line by collecting sequential sections of 1.5 cm from the line 1.0 cm downstream of the inoculation site and further. Each section was cut into halves and the two halves were placed in 1 mL 0.9% saline solution. The samples were then sonicated for 5 min at 45 kHz (VWR Ultrasonic Cleaner USC-THD) and degassed for 2 min at the power setting 6 prior to analysis on the FACS.

Plasmid loss from the biofilm and planktonic samples were detected using FACS with a 488 nm (20 mW) laser connected to a green fluorescence detector (bandpass filter 530/30 nm) and 561 nm (50 mW) laser connected to the red fluorescence detector (bandpass filter 610/20 nm). The BD FACSDiva software v.6.1.3 was used for both operating and analyzing. See Supplementary Fig. 2 for gating strategy.

### Competition assay

A competition assay was performed to measure the fitness effect of the plasmid on the strains. This was done as DelaFuente et al.^25^ with modifications. In brief, overnight cultures of the strains with and without plasmid were washed in LB 3 times and adjusted to OD600 1. The adjusted cultures were then mixed and used to inoculate 5 µL into 5 ml LB. The tubes were incubated at 24 °C at 250 rpm. To estimate the initial and final numbers the adjusted cultures and mixtures were measured on the FACSAria IIIu flow cytometer in 0.9% saline solution (NaCl: 9 g L^−1^). The dilution used for the flow cytometry and sampling time was recorded allowing calculation back to event pr ml based on the input volume taken up by the machine.

### Plasmid loss in alginate beads

Alginate beads were prepared according to the published protocol by Sonderholm et al.^26^, with modifications^27^. Beads were prepared using alginate extracted from the brown alga *Laminaria hyperborea* (Protanal LF10/60; FMC Biopolymer, Drammen, Norway). In total, 100 mL of alginate solution was prepared by suspending 4.9 g of Protanal LF10/60 in 70 mL of MilliQ water at 50 °C until dissolved, and then added 30 mL of MilliQ water. The alginate solution was subsequently autoclaved at 120 °C for 20 min. For bead preparation, 7.5 mL of the alginate solution was mixed with 2.5 mL washed and OD-adjusted cell suspension of pre-grown planktonic cultures. Cell cultures were added to the alginate solution, aiming for e.g., ~10^8^ cells mL^−1^. Droplets of the alginate-bacteria suspension was dispensed via a 21-gauge needle placed 3 cm above the surface of a stirred 0.25 M CaCl_2_ solution at room temperature. Droplets were made at a constant speed using a syringe pump. A total of 2 mL alginate-bacteria suspension was dispensed into droplets yielding beads with an approx. size of 2.4 mm. Beads were allowed to harden for 5 min in the stirred solution before being transferred to a new 250-mL flask with 100 mL of 0.25 M CaCl_2_ hardening. Beads were allowed to fully harden for 30 min while shaking at 150 rpm at room temperature. After hardening, beads were transferred to a new 250-mL flask with 100 mL of a 0.9% NaCl washing solution to remove loosely attached cells from the surface of the beads. Beads were washed in the washing solution for 30 min while shaking at 150 rpm at room temperature. After washing, the beads were transferred to new 250-mL flasks with 100 mL 10% LB and incubated overnight at 24 °C with horizontal shaking at 150 rpm.

Individual beads were selected from the flasks and divided into two equal halves using a razor-blade. One half was placed with the cut-side downward in a Transparent WillCo-dish^®^ Glass Bottom Dish (HBST-12, Willco Wells B.V., Dish/Glass Ø: 35/12 mm, Glass thickness: 0.17 mm/#1.5). The bead was covered with 130 µL 0.9% NaCl as a wetting solution to keep the bead moist. Visualization was performed with a confocal laser scanning microscope (CLSM) (LSM 800, Zeiss) with a EC Plan-Neofluar10x/0.30 M27. Z-stacks were recorded using Axiocam 503 mono, and 488-nm excitation for GFP and 561-nm for mCherry. The free open-source software ImageJ (National Institute of Health, USA) was used to set the threshold using Otsu’s algorithm for the background signal before quantification and distribution analysis in the R statistical language.

### Plasmid loss in biofilm examined by CLSM

Biofilms were grown in flow cells following the same procedure described in Olsen et al.^28^. The flow cells were 1 µ-slide VI^0.4^ (Ibidi) to allow for the visualization of biofilm development. The flow-cell system was filled with LB medium diluted five times and chambers were inoculated with overnight cultures adjusted to an OD_600_ of 0.05. Following inoculation, the bacteria were allowed to settle for 1 h before starting the pump at a flow rate of 2.5 mL h^−1^ at room temperature. Biofilms were analyzed in situ on an inverted CLSM (LSM 800, Zeiss) with a Plan-Apochromat 63x/1.4 oil DIC M27. Z-stacks of different spots were recorded after 48 h. The free open-source software ImageJ (National Institute of Health, USA) was used again to set the threshold for the background signal with Yen and Moments algorithm for the two channels before quantification and distribution analysis in the R statistical language.

### Statistical analysis of FACS data

Samples from planktonic cultures were analyzed by FACS in four biological replicas, which were used to calculate the mean and standard deviation. Samples from biofilm cultures with WT strains and MRB1 with the conjugative plasmid were analyzed by FACS in four biological replicas and three technical replicas and MRB1 with the conjugation-deficient plasmid in three biological replicas and three technical replicas, for which the measurements were averaged. Differences in trends of plasmid loss were compared with linear models and the *emtrends* function from the emmeans package^29^.

### Image analysis of flow-cell biofilms

For each image, the positions in the *z* direction were shifted by subtracting the average *z* position weighted by the number of pixels in each layer; thus, ensuring that a resulting *z* position of 0 denotes the layer with the most biomass, making different images comparable. Pixel counts from different images within each biological replica were averaged. For each biological replica a loess regression was fitted to the plasmid loss proportion. For statistical analysis of data presented in Fig. 2a, the ratio of plasmid-free and plasmid-carrying cells were calculated for each layer of the images in the *z* direction. Next, averages were calculated based on the ratios in the top and bottom of the biofilms. Top and bottom were designated as corrected *z* position above or below 0, respectively, such that each contain half the biomass. Images are available at Zenodo (10.5281/zenodo.5493705).

### Image analysis of alginate beads

Images were the first median smoothed with a 3 × 3 × 3 filter (*smoothIMG* function). Then the centers of the alginate beads were calculated as the median position of all pixels in the image (*center_of_mass* function). Thereafter aggregates were detected by grouping adjacent pixels including diagonals (*clumps* function^30^), and the distances from the aggregates to the center of the bead were calculated. Aggregates smaller than 50 cubic microns were discarded, and aggregates visibly outside of the alginate beads were removed. The distances to the center were normalized for each bead by dividing by the maximum distance. Images are available at Zenodo (10.5281/zenodo.5493705).

### Reporting summary

Further information on research design is available in the Nature Research Reporting Summary linked to this article.

## Supplementary information

Supplementary information

Reporting Summary

## Data Availability

The data that support the findings of this study are available at Zenodo (10.5281/zenodo.5494443).

## Code availability

Images were quantified using the RCon3D package version 1.2.2 (https://github.com/Russel88/RCon3D) in the R statistical language (*quant* function^31^). Scripts used for image analyses are available at Zenodo (10.5281/zenodo.5493705).
