# Supplementary material for: Biofilms can act as plasmid reserves in the absence of plasmid specific selection
Source: NPJ Biofilms Microbiomes. 2021 Oct 7;7:78. doi: 10.1038/s41522-021-00249-w (PMC8497521; doi:10.1038/s41522-021-00249-w)
Supplement: Supplementary file 1 — Supplementary information [file 41522_2021_249_MOESM1_ESM.pdf]

Supplementary Table 1. Strains and plasmids

Strain or plasmid	Relevant characteristic	Source
Strains		
P. putida KT2442	Mt-2 hsdR1 (r^- m^+) Rif^R	Lopez-Sanchez *et al.* (2013)
P. putida MRB1	KT2442 miniTn5::lapG RifR, KanR	Lopez-Sanchez *et al.* (2013)
P. putida KT2442::gfp	Gen^R	This study
P. putida MRB1::gfp	Gen^R	This study
Plasmids		
pMIB4	pKJK5::[Kan^R, $LacI^q$], conjugative, Tet^R	Bahl *et al.* (2007)
pMIB8	pKJK5::[Kan^R, $LacI^q$], conjugation deficient, Tet^R	Bahl *et al.* (2007)
pMIB4-mCherry	pMiB4-Δ*drfA1*::[Gen^R, mCherry]	This study
pMIB8-mCherry	pMiB8-Δ*drfA1*::[Gen^R, mCherry]	This study

References

Lopez-Sanchez, A., Jimenez-Fernandez, A., Calero, P., Gallego, L. D. & Govantes, F. New methods for the isolation and characterization of biofilm-persistent mutants in Pseudomonas putida. Environ. Microbiol. Rep. 5, 679-685 (2013).

Bahl, M. I., Hansen, L. H. & Sørensen, S. J. Impact of conjugal transfer on the stability of IncP-1 plasmid pKJK5 in bacterial populations. FEMS Microbiol. Lett. 266, 250-256 (2007).

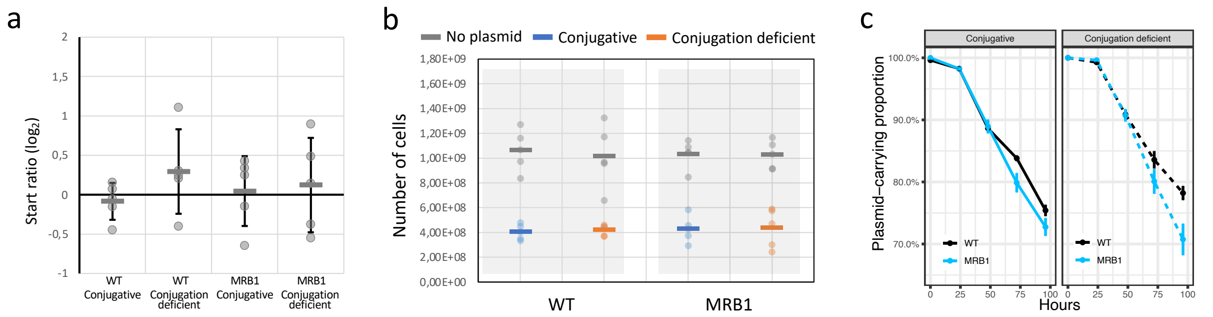

Supplementary Figure 1: Competition between strains with and without plasmids and plasmid loss in planktonic cultures

a) Start ratios of strains with and without plasmids at the beginning of the competition experiment. Vertical bars represent the mean of 5 replicates (dots). There was no significant difference between the start ratios (One-way ANOVA, $p = 0.65$). Error bars are standard deviations. b) Number of cells after competition between strains with and without plasmids for 24h. No significant difference was found between the different strains with plasmids or for strains without plasmids (two-way ANOVA, $p = 0.995$). A significant difference was found when testing strains with plasmids against strains without (two-way ANOVA, $p = 5.53 \times 10^{-15}$). c) Plasmid loss over time in planktonic cultures. Plasmid loss in planktonic cultures of a dispersing (WT) and non-dispersing (MRB1) variant of *P. putida* monitored for 4 days. The conjugation efficiency of the plasmids had no effect on their stability (linear model and the emtrends function, $p = 0.37$ for conjugative and $p = 0.21$ for conjugation deficient plasmid). Error bars are standard error of the mean.

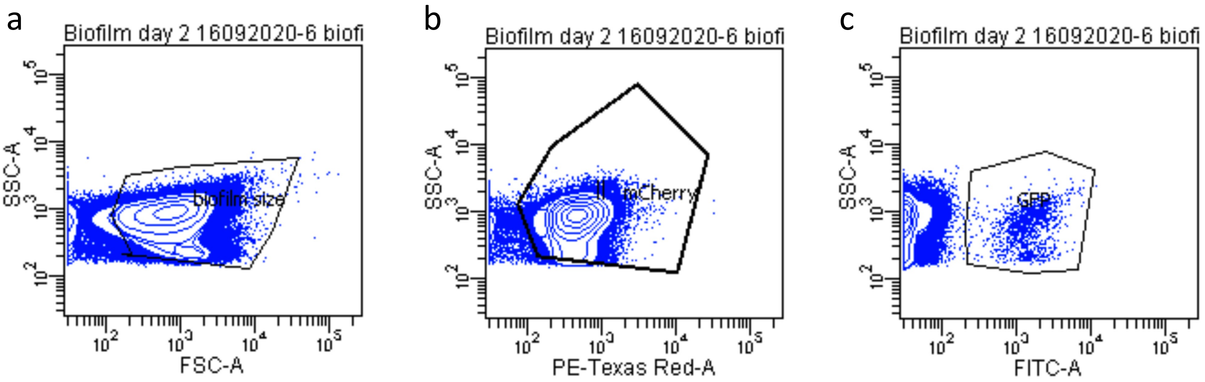

Supplementary Figure 2: Gating strategy for flow cytometry.

Here representative data from biofilm experiments are shown (see also Fig. 1b). a) In SSC/FSC plots, gates were set to record bacterial cells. Red and green fluorescence of the events from the "bacterial" gate were determined by gates illustrated in b) and c), respectively. The "bacterial" gate (a) was set by comparing pure cultures of the used bacterial strains with 0.9% saline solution without cells. Pure cultures of bacterial constructs with GFP, mCherry, and without, were used to determine gates for florescence (b and c).
