# Supplementary material for: Biofilms can act as plasmid reserves in the absence of plasmid specific selection
Source: NPJ Biofilms Microbiomes. 2021 Oct 7;7:78. doi: 10.1038/s41522-021-00249-w (PMC8497521; doi:10.1038/s41522-021-00249-w)
Supplement: Supplementary file 2 — Reporting Summary [file 41522_2021_249_MOESM2_ESM.pdf]

nature portfolio

Corresponding author(s): Mette Burmølle & Jonas Stenløkke Madsen

Last updated by author(s): Sep 10, 2021

Reporting Summary

Nature Portfolio wishes to improve the reproducibility of the work that we publish. This form provides structure for consistency and transparency in reporting. For further information on Nature Portfolio policies, see our Editorial Policies and the Editorial Policy Checklist.

Statistics

For all statistical analyses, confirm that the following items are present in the figure legend, table legend, main text, or Methods section.

n/a	Confirmed	
☐	☒	The exact sample size (*n*) for each experimental group/condition, given as a discrete number and unit of measurement
☐	☒	A statement on whether measurements were taken from distinct samples or whether the same sample was measured repeatedly
☐	☒	The statistical test(s) used AND whether they are one- or two-sided<br>*Only common tests should be described solely by name; describe more complex techniques in the Methods section.*
☐	☒	A description of all covariates tested
☐	☒	A description of any assumptions or corrections, such as tests of normality and adjustment for multiple comparisons
☐	☒	A full description of the statistical parameters including central tendency (e.g. means) or other basic estimates (e.g. regression coefficient) AND variation (e.g. standard deviation) or associated estimates of uncertainty (e.g. confidence intervals)
☐	☒	For null hypothesis testing, the test statistic (e.g. *F*, *t*, *r*) with confidence intervals, effect sizes, degrees of freedom and *P* value noted<br>*Give P values as exact values whenever suitable.*
☒	☐	For Bayesian analysis, information on the choice of priors and Markov chain Monte Carlo settings
☒	☐	For hierarchical and complex designs, identification of the appropriate level for tests and full reporting of outcomes
☒	☐	Estimates of effect sizes (e.g. Cohen's *d*, Pearson's *r*), indicating how they were calculated

Our web collection on statistics for biologists contains articles on many of the points above.

Software and code

Policy information about availability of computer code

Data collection	BD FACSDiva software v.6.1.3<br>ImageJ v1.8.0_172<br>RStudio v1.1.463<br>Zeiss ZEN v2.3
Data analysis	Images were quantified using the RCon3D package version 1.2.2 (https://github.com/Russel88/RCon3D) in the R statistical language (quant function30). Scripts used for image analyses are available at Zenodo (https://doi.org/10.5281/zenodo.5493705).<br>All statistical analysis were performed using the R statistical language. The packages used for specific analysis are described in the manuscript and are all publicly available

For manuscripts utilizing custom algorithms or software that are central to the research but not yet described in published literature, software must be made available to editors and reviewers. We strongly encourage code deposition in a community repository (e.g. GitHub). See the Nature Portfolio guidelines for submitting code & software for further information.

Data

Policy information about availability of data

All manuscripts must include a data availability statement. This statement should provide the following information, where applicable:

- Accession codes, unique identifiers, or web links for publicly available datasets
- A description of any restrictions on data availability
- For clinical datasets or third party data, please ensure that the statement adheres to our policy

The data that support the findings of this study are available at Zenodo (https://doi.org/10.5281/zenodo.5494443).

March 2021

Field-specific reporting

Please select the one below that is the best fit for your research. If you are not sure, read the appropriate sections before making your selection.

☒ Life sciences ☐ Behavioural & social sciences ☐ Ecological, evolutionary & environmental sciences

For a reference copy of the document with all sections, see nature.com/documents/nr-reporting-summary-flat.pdf

Life sciences study design

All studies must disclose on these points even when the disclosure is negative.

Sample size	No sample-size calculations were performed (not relevant)
Data exclusions	No data was excluded
Replication	We replicated each experiment a minimum of three times (as true/biological replicates) and all attempts at replication were successful. Number of technical and biological replicates are specified for each experiment in the manuscript
Randomization	Randomization was not relevant to our study as we compared different bacterial genotypes.
Blinding	Blinding was not relevant to our study as we compared different bacterial genotypes.

Reporting for specific materials, systems and methods

We require information from authors about some types of materials, experimental systems and methods used in many studies. Here, indicate whether each material, system or method listed is relevant to your study. If you are not sure if a list item applies to your research, read the appropriate section before selecting a response.

Materials & experimental systems

n/a	Involved in the study	
☒	☐	Antibodies
☒	☐	Eukaryotic cell lines
☒	☐	Palaeontology and archaeology
☒	☐	Animals and other organisms
☒	☐	Human research participants
☒	☐	Clinical data
☒	☐	Dual use research of concern

Methods

n/a	Involved in the study	
☒	☐	ChIP-seq
☐	☒	Flow cytometry
☒	☐	MRI-based neuroimaging

Flow Cytometry

Plots

Confirm that:

☒ The axis labels state the marker and fluorochrome used (e.g. CD4-FITC).

☒ The axis scales are clearly visible. Include numbers along axes only for bottom left plot of group (a 'group' is an analysis of identical markers).

☒ All plots are contour plots with outliers or pseudocolor plots.

☒ A numerical value for number of cells or percentage (with statistics) is provided.

Methodology

Sample preparation	Bacteria were grown in LB medium from cryo-stocks before experiments were carried out. When required, the growth medium (LB) was supplemented with antibiotics as specified in the manuscript
Instrument	Confocal laser scanning microscope LSM 800 with a EC Plan-Neofluar10x/0.30 M27 (Zeiss)<br>FACSAria IIIu flow cytometer (Becton Dickinson Biosciences).
Software	BD FACSDiva software v.6.1.3
Cell population abundance	In SSC/FSC plots, gates were set to record bacterial cells. Red and green fluorescence of the events from the "bacterial" gate were determined by gates in SSC/PE-Texas Red and SSC/FITC plots, respectively. See also Supplementary Figure 2.

Gating strategy	The "bacterial" gate was set by comparing pure cultures of the used bacterial strains with 0.9% saline solution without cells. Pure cultures of bacterial constructs with GFP, mCherry, and without, were used to determine gates for florescence. See also Supplementary Figure 2

☒ Tick this box to confirm that a figure exemplifying the gating strategy is provided in the Supplementary Information.
